# Parenting Behavior and Early Childhood Mental Health: Cortisol Awakening Response as a Moderator of Child Internalizing and Externalizing

**DOI:** 10.21926/obm.icm.2501011

**Published:** 2025-03-04

**Authors:** Nicole E. Mahrer, Gabrielle R. Rinne, Christine M. Guardino, Danielle A. Swales, Madeleine Ullman Shalowitz, Sharon Landesman Ramey, Christine Dunkel Schetter

**Affiliations:** 1. University of La Verne, Psychology Department, 1950 Third Street La Verne, CA 91750, USA; 2. University of California, Los Angeles, Department of Psychology, 502 Portola Plaza, Los Angeles, CA 90095, USA; 3. Stony Brook University, Department of Psychology, 100 Nicolls Rd, Stony Brook, NY 11794, USA; 4. University of North Carolina at Chapel Hill, Department of Psychiatry, 101 Manning Dr # 1, Chapel Hill, NC 27514, USA; 5. North Shore University Health System Research Institute, 1001 University Pl, Evanston, IL 60201, USA; 6. Fralin Biomedical Research Institute, Virginia Tech, 4 Riverside Circle, Roanoke, VA 24016, USA

**Keywords:** Parenting, cortisol awakening response, early childhood, internalizing, externalizing

## Abstract

Certain observable parenting behaviors contribute to the risk of children developing internalizing and externalizing problems. Yet parenting behaviors do not affect all children uniformly and effects may depend on identifiable child characteristics. One factor is a child’s biological sensitivity to the caregiving environment, an indicator of which is a stress hormone, cortisol. This longitudinal study examines two dimensions of observable parenting behaviors, responsive and rejecting/harsh. These parenting behaviors and child cortisol awakening response (CAR) were measured during home visits in a sample of 100 mostly low-income White and Latina/Hispanic mothers and their children at ages 4-6. Children’s internalizing and externalizing behaviors were assessed one year later. We tested the effects of responsive and harsh/rejecting parenting on child internalizing and externalizing and examined child CAR as a moderator. Results indicated that responsive parenting predicted better child mental health as indexed by fewer internalizing and externalizing behaviors, whereas harsh/rejecting parenting predicted more internalizing behaviors. Harsh/rejecting parenting interacted with child CAR such that harsh/rejecting parenting predicted more externalizing only among children with low CAR; there was no interaction of responsive parenting with child CAR. These results elucidate how child CAR may shape mental health outcomes associated with harsh/rejecting parenting.

## 1. Introduction

Dynamic interactions between parent and child factors are proposed to affect risk for mental health problems in youth. Regulation of child stress physiology, particularly the hypothalamic-pituitary-adrenal (HPA) axis, serves a central role in modifying effects of environmental inputs on child mental health [1, 2], yet the moderating effects of child diurnal cortisol regulation are not well understood.

Cortisol, the key hormonal product of the HPA axis, is secreted in a diurnal pattern throughout the day with a healthy pattern characterized by an increase after waking (cortisol awakening response [CAR]), followed by a gradual decrease across the day and the lowest levels at bedtime [3]. In adults, CAR is characterized by an increase in cortisol (38-75%) in the first hour of waking [4], with the magnitude of CAR hypothesized to be associated with the ability to regulate the body’s resources to manage stress [5]. CAR is observable early in life beginning around age one, but there is some evidence that young children show a blunted, or even negative CAR [6–8]. Furthermore, findings related to CAR and mental health in young children have been mixed. Specifically, in preschool age children, a cross-sectional study [9] found that blunted CAR was related to elevated internalizing problems while a longitudinal study [10] found that elevated CAR predicted internalizing problems. Findings with externalizing problems have been similarly mixed, with no effects of CAR found longitudinally [10] and positive associations between CAR and externalizing found cross-sectionally [9] in younger children.

These mixed findings point to the importance of considering the broader social context when interpreting the adaptive significance of CAR [11]. Emerging evidence suggests that CAR may be an index of environmental sensitivity and closely related to measures of physiological reactivity including cortisol reactivity [12]. Yu et al. [13], for instance, found that CAR moderated the association between neighborhood density and externalizing behaviors in adolescents. A dense neighborhood predicted more externalizing behaviors in adolescents with higher, but not lower, CAR. Yet, in younger samples, two previous studies with other measures of biological sensitivity found that blunted reactivity increased susceptibility to the stress in the environment. Erath and colleagues found a stronger positive association between harsh parenting and externalizing problems among children lower in skin conductance reactivity [2]. Similarly, Somers and colleagues found that maternal postpartum depression was more strongly associated with more infant behavior problems in infants with lower respiratory sinus arrhythmia (RSA) [14]. Parenting behaviors are an integral component of a child’s environment, yet no studies to our knowledge have examined if CAR moderates the effects of parenting behavior on child mental health. Prior studies on the moderating role of diurnal cortisol focused mainly on adolescents [13, 15]. Understanding these processes in younger age groups is important given that this is prior to potential HPA axis recalibration in adolescence [16–18] and can inform effective parenting strategies used with children at this young age. Thus, this study seeks to examine whether the effects of responsive and rejecting/harsh maternal parenting behaviors on internalizing and externalizing behaviors in young children are moderated by child CAR in a diverse community sample of mothers and children recruited for a larger longitudinal study in three regions of the U.S.

## 2. Materials and Methods

### 2.1 Participants and Methods

The study includes 100 Latinx and White mother-child pairs from primarily low-income families (Table 1). All were recruited for the Community Child Health Network research study [19] from three of the five study sites (Washington, D.C., Lake County, IL, and Eastern rural, NC) and followed up as young children for the present study [20]. Mothers and children participated in the study when the child was approximately age 4 (Time 1; *M* = 3.85 years, S.D. = 0.42) and one year later (Time 2; *M* = 5.06 years, S.D. = 0.46). During home visits, trained assessors conducted structured interviews with the mother, videotaped play between the mother and child, and provided standardized instructions/materials for saliva sample collection. The Institutional Review Board at each site approved the study, and mothers provided written informed consent for themselves and their child.

### 2.2 Measures

#### 2.2.1 Responsive and Rejecting/Harsh Parenting

At Time 1, maternal parenting behaviors were coded using the validated 36-month mother-child interaction coding system from the National Institute of Child Development (NICHD) Study of Early Childcare and Youth Development [21] following a video-recorded 15-minute semi-structured play task. Responsive parenting included behaviors demonstrating Sensitivity, Positive Regard, and Stimulation of Cognitive Development. Rejecting/Harsh parenting include behaviors of Intrusivness and Negative Regard (See Supplementary Material for a description of the coding procedure and reliability information).

#### 2.2.2 Child Cortisol Awakening Response

Mothers collected saliva samples from their children immediately after waking, 30 minutes after waking, and at bedtime for three days following the Time 1 visit. Saliva was collected using absorbent Weck-Cel Spears (Beaver-Visitec International, Waltham, MA, USA). Vials were centrifuged at 3000 RPM for 15 mins, and then stored in an −80 degree Celsius ultralow freezer until shipped on dry ice to the Technische Universität Dresden (Kirschbaum, Dresden University of Technology, Germany) for assay (See Supplementary Material for cortisol collection details). Following expert consensus guidelines [22], cortisol values were winsorized to 3 SDs above the mean if higher than that value, and log-transformed to adjust for non-normality prior to calculating the CAR index. CAR was calculated as the difference between cortisol levels at waking and 30 minutes later (CAR values were not calculated if there was more than a 10-minute delay from this prescribed time). The CAR measure used in the current study is an average of the CAR values across the 3 days of data collection. Four children who were taking steroid medication were excluded from the analyses.

#### 2.2.3 Child Mental Health

At Time 2, mothers reported on child mental health symptoms from the past 2 months using the Child Behavior Checklist 1.5-5 years (CBCL; [23], a validated parent-report measure. Standardized t-scores were calculated for two major subscales: *Internalizing* (α = 0.84) and *Externalizing* (α = 0.91). T-scores above 60 are considered at-risk.

#### 2.2.4 Demographics

Mothers reported their age, education, and household income; race and ethnicity for themselves and their child; and child age and sex.

### 2.4 Data Analytic Plan

Analyses were conducted using SPSS [24] and Mplus [25] with full information maximum likelihood to handle missing data. Primary analyses used multiple regression to examine the interactive and main effects of parenting on child internalizing and externalizing, controlling for per capita household income. We probed interactions using simple effects analyses (−1 SD, mean, +1 SD) [26] and Johnson-Neyman analyses (to determine regions of significance) if p < 0.10 [27]. We explored trending interactions to inform future research. We examined main effects of parenting on child internalizing and externalizing behavior if p > 0.10 for interactive terms.

## 3. Results

### 3.1 Descriptive Analyses

Table 1 summarizes demographic characteristics and scores on parenting and child outcome measures. Internalizing problems were in the at-risk or higher range for 20% of the sample and externalizing problems were in the at-risk or higher range for 13% of the sample. Table S1 shows the bivariate correlations among study variables.

### 3.2 Primary Analyses

Table 2 shows results of the multiple regression analyses, described below.

#### 3.2.1 Responsive Parenting

Interaction terms for responsive parenting with CAR were not significant (all p’s > 0.40). Main effects of responsive parenting were significant in that more responsive parenting was associated with lower child internalizing (b = −4.85, β = −0.26, *p* = 0.018, R^2^ = 0.14) and externalizing behaviors (b = −5.31, β = −0.29, *p* = 0.011, R^2^ = 0.09), both small effect sizes.

#### 3.2.2 Rejecting/Harsh Parenting

**Figure 1:**
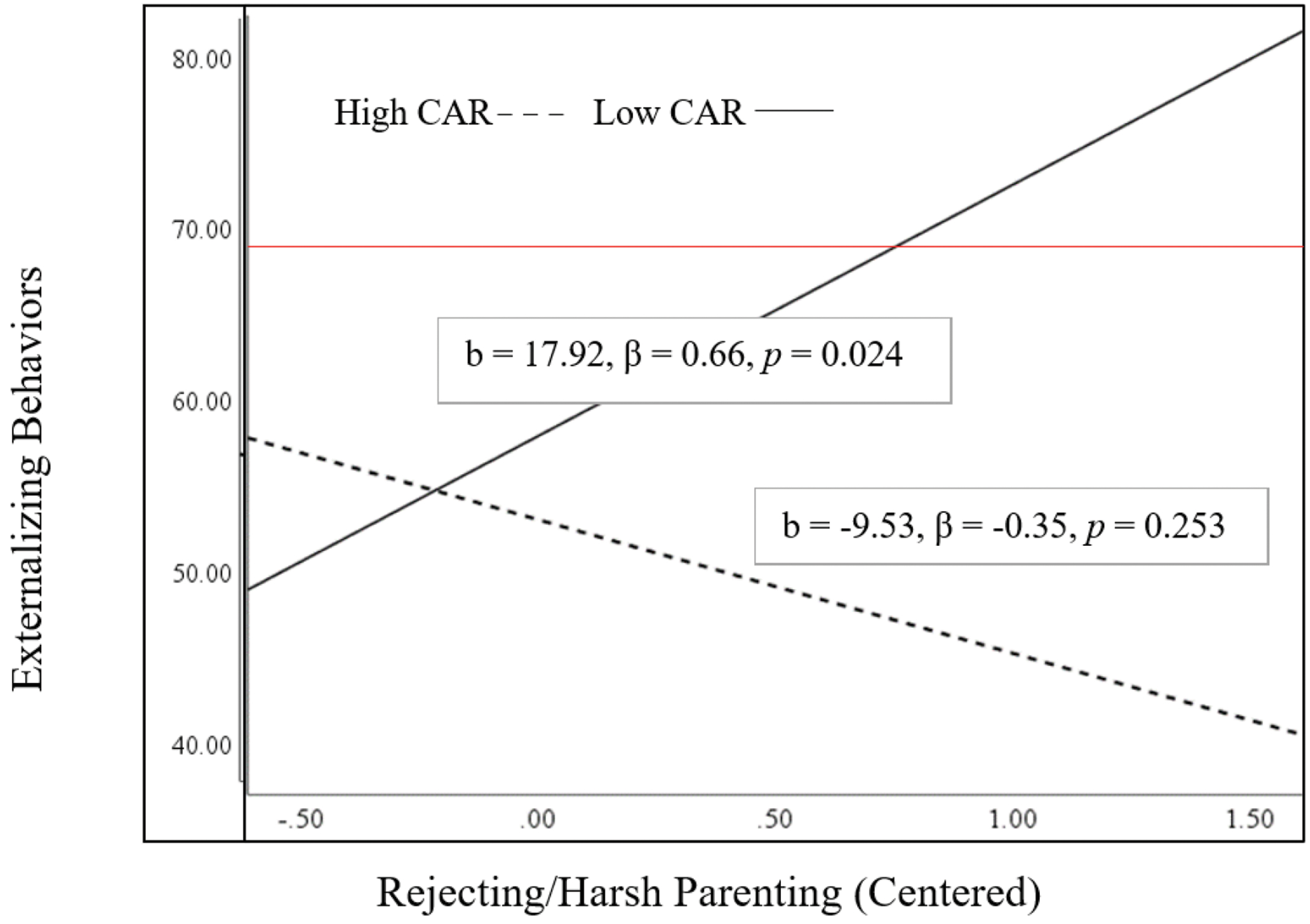
Interaction between Rejecting/Harsh Parenting and Child Cortisol Awakening Response (CAR) Predicting Externalizing Behaviors. Note: Red line indicates at-risk cut-off for externalizing behaviors.

The interaction between rejecting/harsh parenting and child CAR in predicting externalizing behaviors approached significance (b = −18.59, β = −0.30, *p* = 0.057, R^2^ = 0.15), a small effect size. When child CAR was low, rejecting/harsh parenting predicted significantly more child externalizing behaviors (b = 17.92, β = 0.66, *p* = 0.024). This was a moderate-sized effect. However, there was no association when child CAR was high (b = −9.53, β = −0.35, *p* = 0.253; Figure 1). Johnson-Neyman regions of significance showed significant effects of rejecting/harsh parenting on child externalizing when child CAR was 0.27 standard deviations below the mean or lower (i.e., a CAR below −0.014; Figure S1).

Interaction terms for rejecting/harsh parenting and child CAR in predicting internalizing behaviors were not significant; however, there was a significant main effect of rejecting/harsh parenting on child internalizing. More rejecting/harsh parenting was associated with more child internalizing behaviors (b = 7.32, β = 0.27, *p* = 0.028; R^2^ = 0.16), a small effect size.

## 4. Discussion

In a longitudinal study, we examined whether child CAR modified the associations between observed parenting behaviors at one timepoint and parent-reported child mental health approximately one year later in an ethnically diverse, low to middle income sample of mothers and children. Rates of internalizing and externalizing problems in the current sample were comparable to previous studies of mental health in non-referred preschool-aged children [28]. We found that responsive parenting predicted lower child internalizing and externalizing one year later. Rejecting/harsh parenting was associated with more internalizing behaviors regardless of child CAR. However, rejecting/harsh parenting was significantly associated with more externalizing behaviors only in children who had a blunted CAR or who did not mount a CAR. This finding is consistent with the two previous studies referred to above that were conducted with younger samples; both found that blunted reactivity, albeit blunted skin conductance [2] and RSA [14], not cortisol, increased susceptibility to negative parenting environments. Although studies with adolescents have shown higher CAR is associated with greater susceptibility to environmental risk [13], it may be that blunted CAR confers greater susceptibility to environmental risk in younger children, potentially due to pubertal changes that affect the HPA axis [18]. This study contributes to a small but growing literature on diurnal cortisol rhythm as a physiological marker of sensitivity to the environment and to our knowledge, it is the first in early childhood. Future studies with larger childhood samples are needed to replicate the findings in this brief report. Recent work has similarly found that the influence of parenting behaviors on child physiological functioning/outcomes varies depending on child behavior [29]. Together with the present findings, these results highlight the importance of considering joint child and parent contributions to child adjustment.

Prior evidence indicates that rejecting/harsh parenting behaviors and a blunted CAR are individually associated with externalizing behaviors in early childhood [9, 30]. This study is the first to test moderation. The *attenuation hypothesis* posits that individuals with blunted stress responses seek out riskier situations to feel a stronger neurobiological response [31] and *sensation-seeking theory* describes low arousal as an unpleasant physiological state [32]. One argument is that children with lower physiological arousal (e.g., blunted CAR) may seek stimulation by engaging in externalizing behaviors [33]. Physiologically under-aroused children may also be more likely to learn and model coercive and aggressive behaviors from parents because they are less impeded by high physiological arousal [34, 35]. A second line of thought is that children with lower physiological reactivity may have different subjective experiences of harsh punishment (e.g., be less upset by or have poorer ability to learn from harsh punishment) [28]. Fearlessness, failure of avoidance learning, and punishment insensitivity have been linked to other markers of physiological reactivity [36, 37]. Our results fit both of these theoretical explanations. It is important to keep in mind that rejecting and harsh parenting behaviors are less common during an observed free-play task compared to what occurs day-to-day. In the current sample, “rejecting/harsh” types of behaviors looked like the parent being more intrusive or overcontrolling during the play task. For example, if a mother tended to direct the play rather than letting the child take the lead by interrupting the play to correct what the child was doing, or change the toy the child is playing with, or insisting that the child continue to play with an object even if they did not want to.

In this study, child CAR did not modify the association between harsh/rejecting parenting behaviors and internalizing behaviors, nor did CAR values modify the association between responsive parenting and either index of child mental health. *Emotional security theory* states that the goal of a child’s regulatory functioning is to feel secure in their environment [12]. If a child’s actions are consistently dismissed, rejected, or criticized as in harsh and rejecting parenting behaviors, the child’s sense of security may be undermined. This would tend to adversely affect child self-esteem and increase internalizing symptoms [38, 39]. In contrast, responsive parenting as characterized by parental warmth, sensitivity, and positivity promotes a child’s sense of security in their environment and their relationships with caregivers, which in turn may protect against mental health symptoms [12, 40] regardless of child CAR values.

The current sample was recruited in a community-based participatory research study on maternal and child disparities in urban, suburban, and rural regions of the U.S. and had high representation from low-income and Latinx/Hispanic families who are historically underrepresented in research. In addition, the objectively-coded measures of parenting behaviors reduce potential bias, and in-home assessment of parent-child interactions increased ecological validity. However, the current study did not measure child mental health at the first timepoint, limiting causal inferences regarding possible child-elicited, and bidirectional effects. Also, the observed play task, while producing ratings that are more objective than self-report measures, may restrict range in rejecting/harsh parenting behaviors. Further, only half of the sample mounted a significant CAR. Previous CAR studies with younger samples have similarly found flat and even negative sloped CARs [6–8]. However, this could be due to incomplete compliance with the in-home sampling protocol and in general to difficulties collecting diurnal cortisol from young children [41], although cortisol was collected across 3 days and MEMS cap data suggests compliance. Finally, the small sample in this preliminary study precluded correction for Type I error rates. Future studies with larger samples are required to replicate these novel findings.

## 5. Conclusions

These findings are in keeping with the premise that responsive parenting promotes positive child mental health in families of varying ethnicity, of low income, and geographical location, while rejecting/harsh parenting behaviors contribute to less favorable child mental health. The results of the present study also suggest that the effects of mothers’ parenting on child externalizing may be moderated by children’s CAR, a validated index of their diurnal stress response system and likely marker of their susceptibility to the effects of environmental stressors. This seemingly complex relationship between parent and child contributions to children’s mental health should next be examined using data that permit researchers to control for baseline child adjustment in the prediction of subsequent outcomes.

## Supplementary Material

Supplementary Material

## Figures and Tables

**Table 1:** Sample Demographics and Descriptive Statistics (N = 100).

Mother Race/Ethnicity	N (%)	
Latina/Hispanic	60 (60)	
Non-Hispanic White	40 (40)	
Maternal Language Preference (within Hispanic/Latinas)		
English	25 (42)	
Spanish	35 (58)	
Child Sex		
Girls	54 (54)	
Boys	46 (46)	
	M (SD)	Minimum-Maximum

Mother Age (Years at T1)	33.76 (5.46)	24.16-33.76
Household Income Per Capita	$13,573 ($13,155)	$22.08-$51,558.00
Mother Education (Years)	12.57 (3.51)	6-21
Child Age (Years at T1)	3.85 (0.42)	3.46-5.48
Child Age (Years at T2)	5.06 (0.46)	4.31-6.11
Responsive Parenting (T1)	2.87 (0.52)	1.50-3.83
Rejecting/Harsh Parenting (T1)	1.29 (0.36)	1.00-2.50
Child Mean CAR ug/dl (T1)	0.04 (0.20)	−0.396-0.990
Child Internalizing Behaviors (T2)	52.20 (9.72)	29.00-77.00
Child Externalizing Behaviors (T2)	48.89 (9.80)	28.00-71.00

Child Diurnal Cortisol (T1)		
Day 1		
Cortisol waking values (ug/dl)	0.31 (0.25)	0-1.43
Cortisol waking + 30 min values (ug/dl)	0.39 (0.64)	0.02-1.81
CAR (ug/dl)	0.03 (0.23)	−0.396-0.832
Day 2		
Cortisol waking values (ug/dl)	0.29 (0.24)	0.020-1.51
Cortisol waking + 30 min values (ug/dl)	0.34 (0.23)	0.030-1.44
CAR (ug/dl)	0.05 (0.28)	−0.914-0.990
Day 3		
Cortisol waking values (ug/dl)	0.35 (0.48)	0-1.81
Cortisol waking + 30 min values (ug/dl)	0.30 (0.18)	0-0.79
CAR (ug/dl)	−0.02 (0.26)	−1.44-0.380

**Table 2:** Multiple Regression Analyses.

	Externalizing Behaviors	Internalizing Behaviors
	
	β (SE)	β (SE)
Predictors (models separated by bold lines)		
Responsive Parenting	−0.29** (0.11)	−0.26* (0.11)

Rejecting/Harsh Parenting	0.16 (0.13)	0.27* (0.12)

Rejecting/Harsh Parenting	0.14 (0.13)	0.25* (0.12)
CAR	**−0.30**^†^ (0.16)	**−0.28**^†^ (0.15)
Rejecting/Harsh Parenting X CAR	**−0.30**^†^ (0.16)	−0.15 (0.15)

Notes: All analyses controlled for per capita income. Moderation results are shown if the interaction was significant. CAR did not have any main effects on child mental health besides those noted in the table. n. s. not significant;

† p < 0.10;

* p < 0.05;

** p < 0.01.

## Data Availability

The data that support the findings of this study are available from the corresponding author, [NM], upon reasonable request.
