# Supplementary material for: Parenting Behavior and Early Childhood Mental Health: Cortisol Awakening Response as a Moderator of Child Internalizing and Externalizing
Source: OBM Integr Compliment Med. Author manuscript; Available in PMC 2026 Mar 4. (PMC12046604; doi:10.21926/obm.icm.2501011)
Supplement: Supplementary Material [file NIHMS2068592-supplement-Supplementary_Material.pdf]

Supplementary Material

1. Responsive and Rejecting/Harsh Parenting

Maternal parenting behaviors were coded using the validated 36-month mother-child interaction coding system from the National Institute of Child Development (NICHD) Study of Early Childcare and Youth Development (1993) following a video-recorded 15-minute semi-structured play task at Time 1. Mothers and children were offered toy boxes and were instructed to play with the toys for 15 minutes. Mothers engaged with their children in their language of preference (35 Spanish, 65 English). Trained English and Spanish-speaking coders scored parenting behaviors along seven dimensions: (1) *Sensitivity to Nondistress*: being attuned to, centered on the child, letting the child guide play; (2) *Positive Regard*: warmth, praise, physical affection; (3) *Stimulation of Cognitive Development:* efforts to facilitate learning, focus the child's attention on attributes of toys, promote more mature play; (4) *Intrusiveness*: adult-centered, controlling the interaction, imposing the parent's agenda regardless of child signals; (5) *Negative Regard:* maternal anger, frustration, impatience, other indicators of negative regard toward the child; (6) *Detachment:* disengaged in the play or emotionally uninvolved with the child; (7) *Flatness of Affect*: mother not animated in the play session, displaying flat affect in facial gestures and vocal tone. Each parenting dimension was scored numerically on a scale from 1-4, with 4 representing the highest level and 1 the lowest. All coders were blind to other data gathered on study participants. To assess reliability, 24% of the videos were randomly selected and coded independently by an expert coder to obtain inter-rater reliability which ranged from 81% to 100% for the subscales (M = 94%).

Based on exploratory factor analysis with varimax rotation of subscale scores, two composites were derived and scored: Responsive Parenting (Sensitivity, Positive Regard, Stimulation of Cognitive Development) (loadings > 0.611; r's > 0.34, $p < 0.001$) and Rejecting/Harsh Parenting (Intrusiveness, Negative Regard (loadings > 0.72; $r = 0.40$, $p < 0.001$). Maternal Detachment and Flatness of Affect did not load on any factors above 0.3 and were therefore not examined in the current study.

2. Child Cortisol Awakening Response

Mothers collected saliva samples from their children at three time points for three days following the Time 1 visit. They were instructed to take the first sample immediately after the child woke up, the second sample 30 minutes after waking, and the third at bedtime. Saliva was collected using absorbent Weck-Cel Spears (Beaver-Visitec International, Waltham, MA, USA). Instructions noted that children should not eat, drink, or brush teeth before providing a saliva sample. Mothers were asked to write the date and time of collection, refrigerate the samples, and return them by overnight mail in provided mailer along with daily diaries about the child's wake time, bedtime, sample collection times, and any child medications. MEMS track caps (MWV Switzerland Ltd.) logged the sample times for 20% of participants and confirmed accurate reporting. The received vials were centrifuged at 3000 RPM for 15 mins, and then stored in an -80 degree Celsius ultralow freezer until shipped on dry ice to the Technische Universität Dresden (Kirschbaum, Dresden University of Technology, Germany) for assay. Cortisol values were winsorized to 3 SDs above the mean if higher, and log-transformed to adjust for non-normality prior to calculating the CAR index. CAR was calculated as the difference between cortisol levels at waking and 30 minutes later. If the

second sample was collected <20 minutes or >40 minutes after the waking sample, the value that day was excluded from the mean CAR calculation from all days.

Table S1 Bivariate Correlations among Parenting Dimensions and Child Variables.

	Responsive Parenting	Rejecting /Harsh Parenting	Detached Parenting	Flat Affect Parenting	Child CAR	Child Internalizing	Child Externalizing
Responsive Parenting (T1)	1						
Rejecting/Harsh Parenting (T1)	-0.44**	1					
Detached Parenting (T1)	-0.54**	0.18†	1				
Flat Affect Parenting (T1)	-0.44**	0.15	0.28**	1			
Child CAR (T1)	0.34*	-0.11	-0.14	-0.10	1		
Child Internalizing (T2)	-0.34**	0.29*	0.03	-0.03	-0.23†	1	
Child Externalizing (T2)	-0.26*	0.17	0.18	0.12	-0.13	0.62**	1

Note: †p < 0.10, *p < 0.05, **p < 0.01.

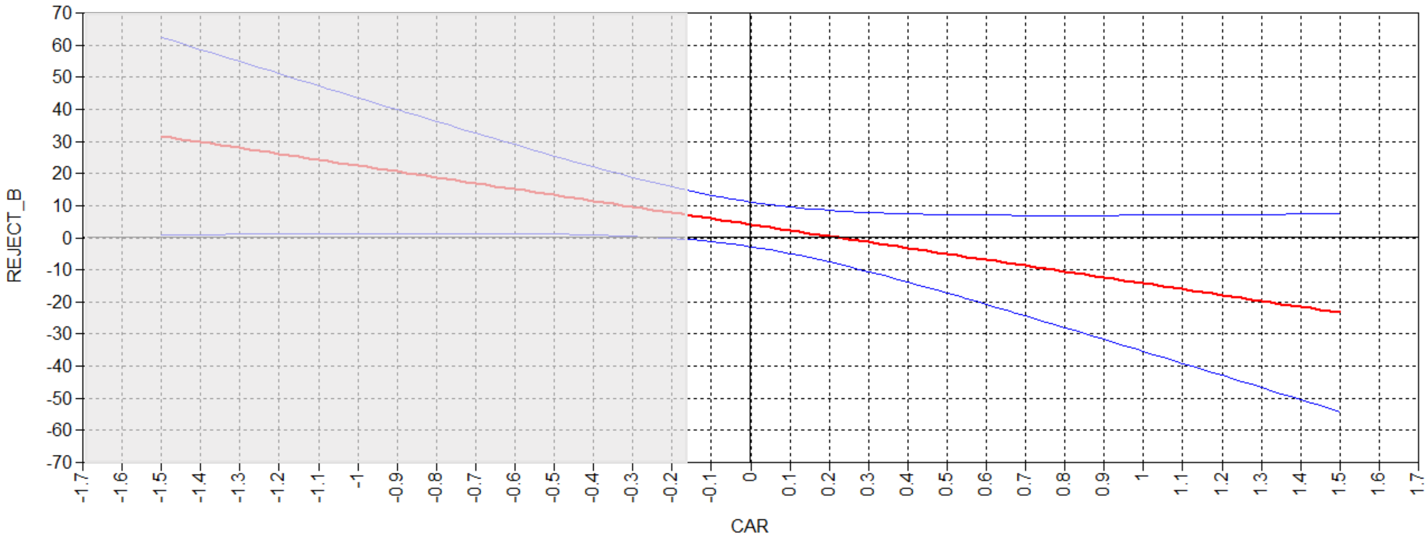

Figure S1 Johnson-Neyman Regions of Significance. Note. Region of significance is highlighted in grey.
